# Performance of an adult Brazilian sample on the Trail Making Test and Stroop Test

**DOI:** 10.1590/S1980-57642014DN81000005

**Published:** 2014

**Authors:** Kenia Repiso Campanholo, Marcos Antunes Romão, Melissa de Almeida Rodrigues Machado, Valéria Trunkl Serrao, Denise Gonçalves Cunha Coutinho, Gláucia Rosana Guerra Benute, Eliane Correa Miotto, Mara Cristina Souza de Lucia

**Affiliations:** 1 Psychology Division - Hospital das Clinicas, University of São Paulo, São Paulo, Brazil; 2 Neurology Department - Hospital das Clínicas, University of São Paulo, São Paulo, Brazil.

**Keywords:** attention, Trail Making Test, Stroop test, demographic analysis

## Abstract

**Objective:**

The Trail Making Test (TMT) and Stroop Test (ST) are attention tests widely used in clinical practice and research. The aim of this study was to provide normative data for the adult Brazilian population and to study the influence of gender, age and education on the TMT parts A and B, and ST cards A, B and C.

**Methods:**

We recruited 1447 healthy subjects aged ≥18 years with an educational level of 0-25 years who were native speakers of Portuguese (Brazilian). The subjects were evaluated by the Matrix Reasoning and Vocabulary subtests of the Wechsler Adult Intelligence Scale-III, along with the TMTA, TMTB and ST A, B and C.

**Results:**

Among the participants, mean intellectual efficiency was 103.20 (SD: 12.0), age 41.0 (SD: 16.4) years and education 11.9 (SD: 5.6) years. There were significant differences between genders on the TMTA (p=0.002), TMTB (p=0.017) and STC (p=0.024). Age showed a positive correlation with all attention tests, whereas education showed a negative correlation. Gender was not found to be significant on the multiple linear regression model, but age and education maintained their interference.

**Conclusion:**

Gender did not have the major impact on attentional tasks observed for age and education, both of which should be considered in the stratification of normative samples.

## Abstract (pt)

**Objetivo:**

Os testes de atenção Trail Making Test (TMT) e Stroop Test (ST) são largamente usados na prática clínica e em pesquisas. O objetivo deste estudo foi fornecer informação normativa para a população brasileira de adultos e estudar a interferência de gênero, idade e educação no TMT parte A e B e no ST cartão A, B e C.

**Métodos:**

Recrutamos 1447 sujeitos saudáveis com idade ≥18 anos, nível educacional de 0-25 anos, falantes nativos do Português (Brasil). Os sujeitos foram avaliados pelos subtestes do Wechsler Adult Intelligence Scale-III Raciocínio Matricial e Vocabulários, além do TMTA, TMTB e ST A, B e C.

**Resultados:**

Entre os participantes a média de eficiência intelectual foi de 103,20 (SD: 12,0), de idade 41,0 (SD: 16,4) anos e de escolaridade 11,9 (SD: 5,6) anos. Houve diferenças significantes por gênero em TMTA (p=0,002), TMTB (p=0,017) e STC (p=0,024). Idade se correlacionou de modo positivo com todos os testes de atenção, enquanto a escolaridade correlacionou-se de modo negativo. Após o modelo de regressão linear múltipla o gênero não manteve correlação significativa, mas idade e escolaridade mantiveram sua interferência.

**Conclusão:**

O gênero não mostrou grande impacto nas tarefas atencionais como a idade e escolaridade que devem, portanto, ser consideradas na estratificação de amostras normativas.

## INTRODUCTION

The concept of attention is associated with the ability to perceive a stimulus, but this is merely one of the aspects related to this cognitive function essential for the functioning of other superior cortical function.^1^

Attention can be defined as a neural mechanism that organizes the input stimuli in our consciousness.^2^ Thus, it enables the processing of information, thoughts or actions relevant for us to function adequately in response to emerging needs. Therefore, it is not hard to understand why many authors refer to attention not only as the climax of mental integration, but as a prerequisite for intellectual manifestation.^3^

Given the multifactorial nature of attention, it can be characterized into three basic forms.^4^ The first of these, sustained attention, represents a state of readiness to detect and respond to a particular stimuli for a period of time. It refers to our ability to maintain a stable response during a repetitive activity. Attention set-shifting is the ability to modify the focus of the attention from one task to another while maintaining fluid behavior, that is, without interrupting the activity. In addition, selective attention refers to the ability to train attention continuously on one stimuli while inhibiting another, therefore, to direct attention to one event over another, where this constitutes an adaptive capacity.^5^

Tests assessing attention are essential in a neuropsychological assessment.^6^ Such instruments include the Trail Making Test (TMT) and the Stroop Test (ST), both widely quoted in international and national studies^7-11^ and considered highly sensitive tasks to lesions in the subcortical region and to frontal lobe lesions and their connections.^6,12^ These tests are therefore measures of executive function and shifting, sustained and selective attention.^6,12^

The TMT first appeared in 1938^13^ and was known as Partington's Pathways. Originally it was divided into two parts, the first, called Part A (TMTA), was used to assess sustained attention and the second, called Part B (TMTB), evaluated attention set-shifting. Currently, other neuropsychological assessment batteries incorporate similar tasks to those proposed by Partington and Leiter,^14,15^ but these instruments are without adaptation or validation for use in Brazil. There is however, national publication of similar tasks in the form of a modified version of the Color Trails Test.^16^

The ST was originally developed by John Ridley Stroop in 1935^17^ to assess selective attention and mental flexibility.^6,12^ Like the TMT, several versions of the ST became available, the most useful of which is the Victoria version.^18^ It was from the Victoria version that Duncan (2006)^8^ published a Brazilian adapted version for use in children from 12 to 14 years of age from public and private schools.

To our knowledge, no investigations on the TMT and ST for native adult and elderly Portuguese (Brazilian) speakers have been published to date. Therefore, the aims of the current study were to investigate the effects of age, education and gender on TMT and ST scores in a sample of Brazilian adults.

## METHODS

**Participants.** The study included 1,447 healthy subjects recruited from the community, associations, schools for adult education, seniors clubs, voluntary or work centers in the five regions of the country, including urban and rural areas, aged18 years or older, with educational level of 0-25 years who were native speakers of Portuguese (Brazilian).

**Procedures.** Subjects who agreed to participate in the study filled out the consent form approved by the research ethics committee of the Hospital das Clinicas of the University of Sao Paulo Medical School (CAPPESq 086/06). Participants were initially interviewed using a semi-structured questionnaire to collect medical and demographic information. The Mini-Mental State Examination (MMSE)^19^ and the Hospital Anxiety and Depression Scale (HADS)^20^ were also administered.

Individuals were excluded if they had previous history of neurological or psychiatric disorders; use of psychotropic drugs; motor, auditory or visual disorders; estimated intelligence quotient (IQ) of less than 80; lower-than-expected scores for education on the MMSE^19^ (20 for illiterates; 25 for 1 to 4 years; 26.5 for 5 to 8 years; 28 for 9 to 11 years, and 29 for higher levels) and score of less than 9 for anxiety and depression as indicated by the HADS.^20^ Consequently, 422 individuals were excluded. Eight for anxiety symptoms, 267 for MMSE scores, 107 for IQ and 40 for inconsistent data.

**Instruments.** The neuropsychological evaluation included the Matrix Reasoning (MR) and Vocabulary from the Wechsler Adult Intelligence Scale-III (WAIS-III)^21^ to obtain the estimated IQ,^22^ the Trail Making Test parts A and B (TMTA and TMTB)^6,13^ and the Stroop Test (ST), adapted Victoria version.^6,8^

The TMT is a task divided into two parts: Part A (TMTA), that requires the connection in ascending order of 25 numbers within circles arranged randomly on an A4 sheet; Part B (TMTB), that requires the connection between 12 letters and 13 numbers in alphabetical and ascending order alternately. Both TMTA and TMTB are preceded by training. The score criterion adopted for the test was the time taken to complete each of the two tasks, but participants who required more than 300 seconds to complete the TMT A or B were not included in the study and classified as having inconsistent data. Errors were corrected promptly by the examiner without stopping the chronometer.^6,13^

The ST followed the guideline specifications suggested by Duncan (2006). Briefly, three cards each containing 24 stimuli against a white background were used. Card A is composed of rectangles printed in green, pink, blue and brown, arranged randomly. Card B, is organized similarly to Card A, but with rectangles replaced by unrelated words to concepts of color (each, never, today and all) printed in uppercase in the 4 colors mentioned. Card C, was also organized similarly to Card A, representing the interference card where the written stimuli were the names of the colors (brown, blue, pink and green), printed in the same colors in such a way that the ink color printed and color name never matched (e.g. brown word printed in pink, green or blue). For the first card, participants have to state the colors of the rectangles as quickly as possible. For cards B and C, subjects must state the color of the printed words and not actually read the words themselves. The criterion score was the time taken to perform the task of each card^6,8^ and all errors were corrected promptly without stopping the chronometer.

**Statistical analysis.** All analyses were conducted using the statistical software package SPSS V20 for Windows V8.1. Continuous and semi-continuous data were analysed initially using the KS-distance test for the evaluation of normality. Consequently, parametric tests were employed. For comparisons of means between genders, Student's t-test was used whereas comparison among age and education groups was performed using ANOVA or Chi-square among frequency comparisons. Pearson's correlation was conducted among attention tests, age and education. Multiple linear regression models were adopted to determine which of these variables had a significant influence on attention tests. Only variables proving significant on the multiple linear regression models were considered for the normative table. The descriptive information was expressed as mean, standard deviation, absolute and relative frequency. A value of p≤0.05 was considered for all results.

## RESULTS

The study included 1025 subjects. Gender, age and education data are showed in Table 1. The intellectual efficiency ranged from 81 to 140 (Table 1). On the mood assessment, subjects scored a mean (SD) of 4.66 (2.1) points for anxiety and 3.37 (2.2) for depression, confirming an absence of these symptoms.^20^ On the MMSE screening instrument, subjects scored a mean of 28.83 (1.3) points, all suggestive of preserved cognition.

**Table 1:** Mean, standard deviation, absolute and relative frequency of sociodemographic data and IQ for all participants and for gender, age and education groups.

		Male (%)	Female (%)	AgeM (SD)	EducationM (SD)	IQM (SD)
All		N=33533	N=69067	N=102541.0 (16.4)	N=102511.9 (5.6)	N=1025103.2 (12.0)
Gender	Female	-	-	N=69042.6 (17.0)	N=69011.85 (5.6)	N=690102.97 (11.6)
	Male	-	-	N=33537.9 (15.0)	N=33512.04 (5.7)	N=335103.85 (12.2)
p				<0.001*	0.610	0.880
Age (years)	18-29	N=11937.1	N=20262.9	N=32124.26 (3.4)	N=32112.62 (5.3)	N=321103.80 (12.1)
	30-39	N=9639.7	N=14660.3	N=24833.88 (3.1)	N=24813.19 (5.6)	N=248102.02 (11.6)
	40-49	N=4929.9	N=11570.1	N=16844.79 (2.8)	N=16812.32 (5.6)	N=168105.58 (12.5)
	50-59	N=3731.4	N=8168.6	N=11854.18 (2.8)	N=11812.25 (5.3)	N=118104.59 (11.2)
	60-69	N=1816.7	N=9083.3	N=10864.19 (2.7)	N=1089.19 (5.0)	N=108102.47 (12.1)
	>70	N=1622.2	N=5677.8	N=7275.44 (4.4)	N=726.67 (4.6)	N=7297.43 (10.2)
p		<0.001*	<0.001*	<0.001*	<0.001*	<0.001*
Education (years)	0-4	N=4630.3	N=10669.7	N=15240.06 (20.7)	N=1523.47 (0.8)	N=15291.5 (8.8)
	5-8	N=645.8	N=11564.2	N=17943.66 (17.54)	N=1796.84 (1.3)	N=17997.08 (10.8)
	9-12	N=7029.2	N=17070.8	N=25041.16 (15.9)	N=25010.76 (0.8)	N=250103.20 (11.2)
	>13	N=15534.1	N=29965.9	N=45437.24 (13.2)	N=45417.33 (2.6)	N=454109.47 (9.3)
p		<0.001*	<0.001*	<0.001*	<0.001*	<0.001*

IQ: Intelligence Quotient; TMTA: Trail Making Test Part A; TMTB: Traill Making Test Part B; STA: Stroop Test Card A; STB: Stroop Test Card B; STC: Stroop Test Card C.

As regards to attention tests, significant differences between genders were observed on the TMTA [female 40.52 (18.6) and male 36.61 (18.2); p=0.002], TMTB [female 87.99 (50.3) and male 80.14 (45.4); p=0.017] and STC [female 29.64(12.7) and male 27.79 (11.4); p=0.024], but not for the STA [female 15.75 (5.1) and male 15.60 (5.1), p=0.660] and STB [female 18.87 (6.7) and male 18.82 (7.1), p=0.912]. The data showed poorer outcomes among women.

Age showed a positive correlation with attention tests [TMTA r=0.432 and p<0.001; TMTB r=0.473 p<0.001; STA r=0.337, p<0.001; STB r=0.485, p<0.001and STC r=0.529, p<0.001], whereas education showed a negative correlation [TMTA r= -0.479 and p<0.001; TMTB r= -0.544 p<0.001; STA r= -0.436, p<0.001; STB r= -0.526, p<0.001and STC r= -0.476, p<0.001]. Thus, considering the influence of these variables on tests of attention, multivariate analysis models were created to investigate which variables were most relevant.

Gender was not found to be significant on the model, but age and education maintained their interference. Older participants needed more time to complete tasks while education showed neuroprotective effects (Table 2).

**Table 2:** Regression Model controlling for age, gender and education on attention tests.

Model		Unstandardized coefficients		t	p
		B	Standard error		
TMTA	(Constant)	45.073	2.423	18.601	<0.001*
	Age	3.794	0.313	12.104	<0.001*
	Gender	1.833	1.031	1.778	0.076
	Education	-6.392	0.453	-14.109	<0.001*
TMTB	(Constant)	117.916	5.903	19.977	<0.001*
	Age	10.488	0.761	13.783	<0.001*
	Gender	2.138	2.499	0.856	0.392
	Education	-21.383	1.105	-19.353	<0.001*
STA	(Constant)	19.071	0.694	27.474	<0.001*
	Age	0.918	0.091	10.126	<0.001*
	Gender	-0.329	0.296	-1.110	0.267
	Education	-1.774	0.130	-13.648	<0.001*
STB	(Constant)	24.028	0.820	29.291	<0.001*
	Age	1.602	0.107	14.959	<0.001*
	Gender	-0.768	0.350	-2.194	0.028
	Education	-2.745	0.154	-17.873	<0.001*
STC	(Constant)	32.369	1.490	21.724	<0.001*
	Age	3.330	0.194	17.122	<0.001*
	Gender	0.202	0.636	0.317	0.751
	Education	-4.223	0.279	-15.137	<0.001*

* Statistical Significance (p≤0.05). p: value of Statistical Significance; TMTA: Trail Making Test Part A; TMTB: Trail Making Test Part B; STA: Stroop Test Card A; STB: Stroop Test Card B; STC: Stroop Test Card C.

Given that both age and education proved significant on the model, these variables were considered in the normative table. Table 3 shows the means and standard deviations of the total scores (in seconds) for the TMTA, TMTB, STA, STB and STC applied to subjects.

**Table 3:** Means and Standard Deviation for TMT and ST scores (seconds) according to age and years of education.

	Age group (years)	Education (years)														
		0-4				5-8				9-12				>13		
		N	M	SD		N	M	SD		N	M	SD		N	M	SD
TMTA	18-29	40	38.06	20.9		49	37.73	15.2		61	34.57	9.1		170	29.63	9.1
	30-39	22	49.96	12.0		36	40.25	16.1		60	35.36	10.7		129	30.92	11.6
	40-49	11	62.65	20.4		33	54.16	23.9		52	34.71	11.8		69	30.81	9.6
	50-59	10	52.40	36.8		19	43.54	19.0		41	37.00	10.1		48	37.46	11.0
	60-69	31	63.42	26.9		22	54.84	16.1		25	44.20	13.9		29	40.59	11.8
	>70	35	75.66	30.9		18	55.78	9.2		11	59.09	16.8		8	44.75	12.8
TMTB	18-29	40	98.06	50.8		48	83.44	39.3		58	70.90	27.5		170	56.97	20.8
	30-39	19	125.68	45.9		35	113.57	37.3		59	69.58	26.3		127	55.49	18.1
	40-49	10	149.30	66.2		29	105.48	52.3		51	73.76	32.5		67	64.42	21.6
	50-59	9	88.67	48.4		18	86.35	34.9		41	79.69	26.2		48	76.58	24.0
	60-69	31	173.03	67.3		21	138.14	51.2		25	100.84	43.7		29	91.14	30.0
	>70	34	191.65	57.0		17	143.18	53.0		10	130.30	41.3		8	94.50	18.1
STA	18-29	40	16.95	6.2		49	16.16	5.3		61	13.11	2.7		170	12.93	2.4
	30-39	23	20.70	6.7		36	15.53	4.1		60	14.34	3.7		129	13.35	2.9
	40-49	13	22.48	5.3		34	19.12	6.3		52	14.14	2.7		69	14.77	3.6
	50-59	10	22.60	6.2		19	18.25	6.4		41	15.55	5.5		48	15.23	3.5
	60-69	31	21.03	6.5		23	18.05	5.9		25	16.74	4.6		29	15.91	3.1
	>70	35	22.66	5.9		16	21.50	7.8		11	20.69	5.1		8	17.70	5.0
STB	18-29	40	19.68	7.6		49	19.69	5.9		61	15.89	4.0		171	14.19	2.5
	30-39	23	25.60	6.6		36	18.78	4.2		60	17.91	5.7		129	14.76	3.1
	40-49	13	28.54	8.8		34	22.63	5.1		52	15.94	3.5		69	17.05	3.9
	50-59	10	30.40	4.8		19	23.40	7.3		41	19.54	7.4		48	18.10	3.8
	60-69	31	26.77	6.5		23	25.48	7.6		25	21.59	4.4		29	18.86	2.7
	>70	35	30.09	8.3		16	28.50	9.6		11	28.18	7.1		8	22.08	7.0
STC	18-29	40	27.61	9.8		49	32.06	11.9		61	22.02	6.1		171	20.40	4.7
	30-39	23	36.13	10.8		36	32.56	11.5		60	27.68	8.7		129	21.90	5.5
	40-49	13	37.62	12.4		34	34.06	8.4		52	25.67	5.2		69	25.99	7.3
	50-59	10	45.40	7.7		19	34.46	9.3		41	31.76	11.1		48	27.54	7.5
	60-69	31	40.16	13.0		23	39.93	10.3		25	37.71	9.9		29	31.12	8.2
	>70	35	53.43	19.6		16	49.21	21.7		11	44.27	13.8		8	39.37	15.4

TMTA: Traill Making Test Part A; TMTB: Traill Making Test Part B; STA: Stroop Test Card A; STB: Stroop Test Card B; STC: Stroop Test Card C.

## DISCUSSION

The aims of the current study were to investigate the effects of age, education and gender on TMT and ST scores in a sample of Brazilian adults. The analysis of sociodemographic variables showed the effect of age and educational level of the individuals on normative data.

Our initial analysis by gender showed that men presented better outcomes than women on the attention tests but were significantly younger than the women. In the literature, there is no consensus regarding influence of gender on attention, where some studies had similar results to the present study,^23,24^ while others showed the opposite^25,26^ or demonstrated little influence of gender on performance.^27-29^

For both instruments, older age was associated with longer execution time, a finding consistent with earlier studies showing lower processing speed is correlated with older age.^30,32^ Similar findings were reported in Korean^23,31^ Greek,^25^ French,^28^ Dutch^26,33^ American,^30^ and Portuguese^34^ populations.

Regarding education, our study demonstrated that lower educational level was linked to longer execution time for the tasks, corroborating the results of other studies.^23,24,31,35,36^

After controlling for these two variables, we observed that the influence of gender did not persist. However, we found that age and education continued to correlate negatively and positively, respectively, with performance on the attention tests. Therefore, even with aging, education continues to exert an important neuroprotective effect. These results are supported by theories of cognitive reserve in previous studies confirming that subjects with greater education suffer less impact in terms of cognitive decline.^37,38^

Hence, based on our findings, we can conclude that gender did not exert a major influence on the proposed attention tasks, while age and education showed significant correlations with performance. These findings demonstrate the importance of carrying out normative studies that are both culture and language-specific, using large samples of individuals of different ages and educational levels. The current results suggest that the use of the TMT and ST might be more appropriate for clinical application in populations with higher levels of education. Nevertheless, future studies should confirm the clinical validity of these measures in patient populations.
